# Antipyretic effect of ibuprofen in Gabonese children with uncomplicated falciparum malaria: a randomized, double-blind, placebo-controlled trial

**DOI:** 10.1186/1475-2875-7-91

**Published:** 2008-05-26

**Authors:** Pierre-Blaise Matsiégui, Michel A Missinou, Magdalena Necek, Elie Mavoungou, Saadou Issifou, Bertrand Lell, Peter G Kremsner

**Affiliations:** 1 Medical Research Unit, Albert Schweitzer Hospital, Lambaréné, Gabon; 2 Department of Parasitology, Institute of Tropical Medicine, University of Tübingen, Wilhelmstrasse 27, 72074 Tuebingen, Germany

## Abstract

**Background:**

Antipyretic drugs are widely used in children with fever, though there is a controversy about the benefit of reducing fever in children with malaria. In order to assess the effect of ibuprofen on fever compared to placebo in children with uncomplicated *Plasmodium falciparum *malaria in Gabon, a randomized double blind placebo controlled trial, was designed.

**Methods:**

Fifty children between two and seven years of age with uncomplicated malaria were included in the study. For the treatment of fever, all patients "received" mechanical treatment when the temperature rose above 37.5°C. In addition to the mechanical treatment, continuous fanning and cooling blanket, patients were assigned randomly to receive ibuprofen (7 mg/kg body weight, every eight hours) or placebo.

**Results:**

The fever clearance time using a fever threshold of 37.5°C was similar in children receiving ibuprofen compared to those receiving placebo. The difference was also not statistically significant using a fever threshold of 37.8°C or 38.0°C. However, the fever time and the area under the fever curve were significantly smaller in the ibuprofen group compared to the placebo group.

**Conclusion:**

Ibuprofen is effective in reducing the time with fever. The effect on fever clearance is less obvious and depends on definition of the fever threshold.

**Trial registration:**

The trial registration number is: NCT00167713

## Background

Fever represents the most apparent clinical manifestation of *Plasmodium falciparum *malaria. The role of fever in defence against malaria or in other infectious diseases remains a matter of debate. However, it has been shown that febrile temperatures inhibit the growth of *P. falciparum in vitro *[1,2].

To control fever, the World Health Organization recommends mechanical measures such as fanning, tepid spoging, and cooling blankets [3]. However, antipyretic drugs are commonly and widely used to treat malarial fever in endemic areas, though there is a controversy about the benefit of reducing fever in children with malaria [4]. Data from Gabon have revealed that neither paracetamol, nor naproxen or metamizol – antipyretics often used in this area – had an effect on fever clearance time [5,6]. Naproxen showed a weak effect in reducing fever peaks and in reducing the time spent with fever [6]. Worryingly, paracetamol increased parasite-clearance times (i.e. inhibited clearance of parasites) and significantly decreased the production of oxygen radicals and tumor necrosis factor, mechanisms of the innate immune response pivotal to combat infections [5].

Another antipyretic drug often used to treat malarial fever in endemic areas is the non-steroidal anti-inflammatory agent ibuprofen. In various studies, ibuprofen has been shown to be more efficacious than acetaminophen (paracetamol) in lowering febrile temperatures of infectious origin in children [7-11]. But the rationale of its use and its capacity of reducing fever due to *P. falciparum *malaria has never been proven in a double blind, placebo controlled trial. In a randomized, double-blind study in Thai adults, comparing a single dose of ibuprofen with paracetamol for the treatment of fever due to uncomplicated falciparum malaria, ibuprofen was significantly more effective than paracetamol in lowering temperatures throughout the first 4.5 h after dosing [12], whereas in Malawian children aged less than five years both drugs were equally effective in reducing fever [13].

In this double-blind randomized controlled trial, the effect of ibuprofen on fever compared to placebo in children with uncomplicated *P. falciparum *malaria in Gabon was investigated.

## Patients and methods

### Study area

The study was conducted at the Medical Research Unit of the Albert Schweitzer Hospital in Lambaréné, Gabon. Malaria is hyperendemic in this area with perennial transmission and an entomological inoculation rate of about 50 infective bites/person/year [14,15].

### Eligibility criteria

Hospitalized pediatric patients (male and female) with clinically uncomplicated falciparum malaria were included in the study. The inclusion criteria were: two to seven years of age, asexual parasitaemia between 20,000 and 200,000/μL, no mixed plasmodial infection, fever with temperature above 38°C or history of fever during the preceding 24 hours, informed consent by parents or guardians. The exclusion criteria were: effective anti-malarial treatment for the present attack (negative urine tests for chloroquine, quinine and sulphonamides (Wilson and Edeson tests, Lignin test)), antipyretic use within six hours of presentation, contraindications to the use of ibuprofen (history of asthma, dyspeptic symptoms, gastro-intestinal bleeding, or allergy to ibuprofen), haemoglobin < 7 g/dl, packed-cell volume < 20%, white cell count > 16,000/μL, platelet count < 40,000/μL, schizontaemia > 50/μL, impaired consciousness, convulsions or history of convulsions, concomitant diseases masking assessment of response.

### Interventions

Patients were hospitalized until two consecutive thick blood smears were negative for malaria parasites. For malaria treatment, all patients received an infusion of 250 mL of 5% glucose with 12 mg/kg of quinine dihydrochloride every 12 h for 72 h, then a single dose of oral sulphadoxine/pyrimethamine (500 mg/25 mg tablet) was given according to weight category (body weight ≤ 10.0 kg: 0.25 tablet; 10.1–14.0 kg: 0.5 tablet; 14.1–20.0 kg: 1.0 tablet; 20.1–30.0 kg: 1.5 tablets) after the last infusion. This regimen has been found to be efficacious in the study area [16].

For the treatment of fever, all patients received mechanical treatment (continuous fanning and cooling blanket) when the temperature rose above 37.5°C. In addition to the mechanical treatment, patient were assigned randomly to receive ibuprofen or placebo. Ibuprofen was provided as syrup of Nurofen^® ^(Boots Healthcare International, Nottingham, UK), 5 mL of syrup Nurofen^® ^containing 100 mg of ibuprofen. The dosage was 7 mg/kg 8 hourly until fever and parasite clearance. Placebo, identical to the test drug in colour, smell and packaging, was given every 8 hours until fever and parasite clearance. The drugs were administered under supervision. In the event of vomiting within one hour of drug ingestion, the dose was immediately re-administered. Placebo was provided by the University Pharmacy, University of Tübingen, Tübingen, Germany.

### Study design

The study was designed as a double blind, placebo controlled study to compare the antipyretic efficacy of ibuprofen versus placebo in children with uncomplicated falciparum malaria.

### Outcome measures

The study primary endpoint was the fever clearance time (FCT) in hours at a fever threshold of 37.5°C. The Fever time (FT) and the Area Under the Curve with fever (AUC_fever_) were used as supportive primary efficacy endpoints. The following definitions for efficacy measures were used:

- Fever clearance times: time from the start of ibuprofen or placebo therapy to the time that the temperature fell below and remained less or equal 37.5°C and remained at ≤ 37.5°C until parasite clearance (two consecutive negative thick blood smears for asexual *P. falciparum*). The FCT was also calculated for fever thresholds of 37.8°C and 38.0°C.

- Fever time: duration in hours that an individual's temperature was above an indicated fever threshold.

- Area Under the Curve with fever: area covered by the temperatures' curve during the hospitalization period (area between the temperatures' curves and an horizontal line which represents different fever thresholds).

The following approaches have been used for the calculation of the FCT and FT: i) a temperature elevations more than 12 hours after parasite clearance were not considered; ii) if at least 12 consecutive temperature values were missing, the patient was considered not evaluable; iii) in the case that no fever was present at baseline and throughout the hospitalization period, the FCT equaled zero.

The secondary endpoints were: i) parasite clearance times, calculated from the beginning of antipyretic treatment to the time of the first of two consecutive negative films for asexual parasites; ii) assessment of adverse events. An adverse event was defined as any sign or symptom that appears or that increases in intensity after the beginning of the antipyretic treatment. A serious adverse event was defined as any untoward medical occurrence that is life threatening or that requires prolongation of hospitalization. The study physicians recorded all adverse events and classified them according to their seriousness, intensity, and causality.

Digital thermometers were used to measure rectal temperature on admission and every hour for the duration of the hospitalization period. Blood smears for parasitaemia were taken at the time of admission and every 8 hours until two consecutive blood smears were negative for asexual *P. falciparum*. The parasitaemia was determined, as described previously [17], from 10 μL of capillary blood distributed evenly over an area of 18 × 10 mm on a glass slide and prepared as a Giemsa-stained thick smear. The number of parasites per 100 oil-immersion fields (1,000×) was counted and multiplied by 600 (using an Olympus CH30 microscope) to calculate the number of parasites per μL of blood. Each blood film was read independently by two experienced laboratory technicians. Physical examination was done prior to treatment and once daily until discharge, and symptom histories was obtained every eight hours. Blood for haematology (haemoglobin, haematocrit, total and differential white cell count and platelet count) was obtained prior to treatment and at the time of discharge.

### Sample size

Sample size calculation was based on fever clearance time (FCT). The difference in mean FCT between the two groups (ibuprofen versus placebo) was estimated from previous studies [5,6]. The sample size was calculated on the assumption of difference in FCT of 15 h between the two groups with standard deviation (SD) of mean FCT of 15 h at a significance level of 5% and a power of 90%. The estimated number of patients needed per group was 22. To allow for patient loss and non evaluable patients 25 patients were included in each group. A total of 50 patients were included.

### Randomization procedures

The patients were allocated by the study physicians to ibuprofen or placebo in the order of admission according to a computer generated randomization list. All study personnel and participants were blinded to treatment assignment for the duration of the study (double blind design). The code was contained in sealed envelopes, each with the study number writing on the outside. The code was kept in a locked cupboard in the hospital in Lambaréné and only the principal investigator and one subinvestigator had direct access to the key. A second set of the code was kept by the University Pharmacy, Tübingen, Germany) where the placebos were manufactured. The code was shown to the investigators once recruitment, laboratory analyses, data collection and data cleaning were complete.

### Statistical methods

Descriptive statistics were used to summarize the baseline data. The analysis for the primary endpoint was per protocol analysis and was based on all evaluable patients. The safety analysis included adverse events from all subjects who received at least one dose of the study drug (intention-to-treat population). Differences between treatment groups were assessed by student t-test. The Area Under the Curve for fever was calculated by use of the trapezoid rule and performed using JMP software (Version 5, SAS Institute, USA). P-values of less than 0.05 were considered significant.

### Ethical aspects

Informed consent procedures, documentation and clinical activities followed Good Clinical Practice guidelines. The ethics committee of the International Foundation of the Albert Schweitzer Hospital in Lambaréné approved the study and informed consent was obtained from each participant's parent or guardian before any study specific procedure was started.

### Trial registration

The present study was registered on 11^th ^September 2005 on the following online registry: clinicaltrials.gov The registration number is: NCT00167713.

## Results

**Figure 1:**
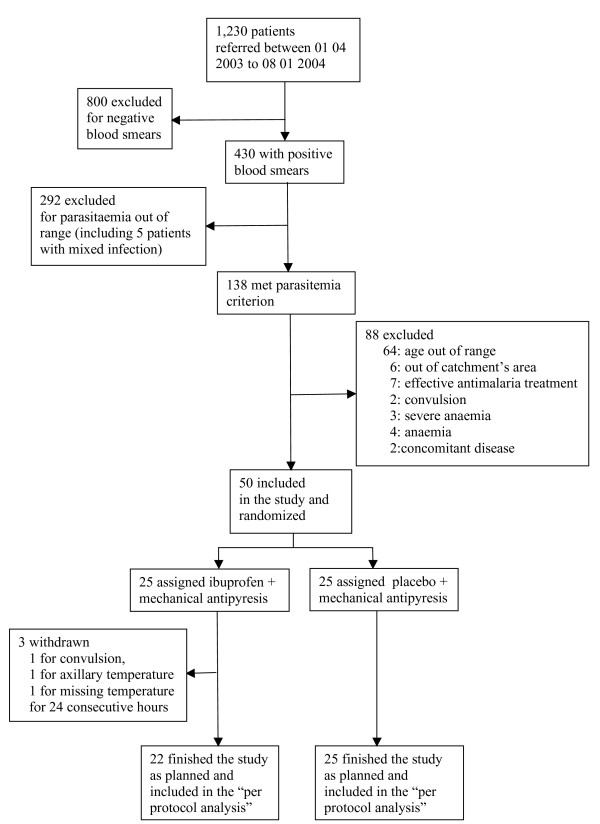


Between April 2003 and January 2004, 1230 children were prescreened for malaria and 430 had a positive blood smear (Figure 1). Fifty children met the inclusion criteria and were randomly assigned to treatment and were analysed. Table 1 shows the baseline characteristics of the patients.

**Table 1:** Clinical, parasitological, and laboratory characteristics on admission

	Ibuprofen (n = 25)	Placebo (n = 25)
**Patients' data**		
Gender (F/M)	10/15	8/17
Age (years)	3.6 ± 1.4	4.5 ± 1.7
Temperature * (°C)	38.1 ± 1.0	38.7 ± 1.1

**Laboratory data**		
Geometric mean parasitemia (range)	63,557 (20,000 – 200,000)	70,516 (20,000 – 200,000)
Hemoglobin (g/dL)	9.3 ± 1.2	9.6 ± 1.3
Packed-cell volume (%)	28.8 ± 4.2	29.6 ± 4.2
Leucocytes count (/μL)	7,400 ± 3,400	7.9 ± 2,300
Platelets (/μL)	157,000 ± 63,000	141,000 ± 43,000

Data were summarized by use of descriptive statistics.

Numbers are given as mean ± SD, unless specified otherwise.

* N = 49 (one patient in ibuprofen group was excluded because axillary temperature was recorded)

Forty-nine patients were hospitalized until fever clearance and two consecutive negative blood smears for malaria parasites. One patient, receiving ibuprofen, had convulsions 12 h after the start of the antipyretic and quinine treatment, and was excluded from the study. Two children, both in the ibuprofen group, were excluded for protocol violation: one for axillary temperature measurement, one for consecutive missing temperature values for 24 hours. Forty-seven children (22 in the ibuprofen group and 25 in the placebo group) completed the study protocol and were analysed for the primary outcome (fever clearance time).

The two groups were similar with respect to baseline characteristics (Table 1).

Patients were hospitalized for a mean (± SD) of 80.0 h (± 10.0) vs. 79.0 h (± 11.0) for ibuprofen and placebo, respectively. Overall, the FCT was significantly lower than the PCT (47.0 h (± 23) vs. 66.0 h (± 15), respectively, p < 0.001).

In Table 2, the two groups are compared with respect to FCT, FT, and AUC_fever _at different fever tresholds. The FCT at fever threshold of 37.5°C was lower in children receiving ibuprofen in addition to the mechanical treatment (41.0 h ± 21) than in those receiving only mechanical treatment (placebo group) (52.0 h ± 24); however, this difference was not significant. Similar results were found for FCT using a fever threshold of 37.8°C (37.0 h ± 21 vs. 44 h ± 24, for ibuprofen and placebo, respectively). Similarily, the difference in the FCT between the two groups using a fever threshold of 38.0 was also not statistically significant (29.0 h ± 23 vs. 41 h ± 22, for ibuprofen and placebo, respectively). However, the measurements of fever time (FT) at different fever thresholds showed that children in ibuprofen group spent significantly less time with fever compared to children in placebo group. Likewise, the AUC_fever _were also significantly lower for the ibuprofen group compared to the placebo group over a wide range of fever thresholds (Table 2). There was no difference in parasite clearance time between the two groups.

**Table 2:** Fever clearance time, fever time, and the area under the curve at different fever threshold in the two treatment groups

	Fever threshold (°C)	Ibuprofen N = 22	Placebo N = 25	Difference^1 ^(95% CI)	P value^2^
FCT^3^, mean (SD)	37.5	41.0 (21.0)	52.0 (24.0)	11.0 (-2.2 – 24.1)	0.102
	37.8	37.0 (21.0)	44.0 (24.1)	7.0 (-6.4 – 20.0)	0.307
	38.0	29.0 (23.0)	41.0 (22.3)	12.0 (-0.9 – 25.5)	0.067

FT^4^, mean (SD)	37.5	18.3 (15.5)	28.0 (16.0)	9.7 (0.5 – 19.0)	0.040
	37.8	13.4 (13.4)	22.6 (13.1)	9.2 (1.4 – 17.0)	0.022
	38.0	11.4 (11.9)	19.9 (11.5)	8.5 (1.6 – 15.3)	0.017
	38.5	7.4 (8.5)	13.0 (9.1)	5.7 (0.5 – 10.9)	0.032
	39.0	4.2 (5.2)	9.1 (7.8)	4.8 (0.9 – 8.7)	0.018
	39.5	1.8 (2.6)	6.0 (6.6)	4.2 (1.2 – 7.2)	0.007
	40.0	0.2 (0.4)	2.5 (3.9)	2.3 (0.7 – 4.0)	0.007

AUC_fever_^5^, mean (SD)	37.5	16.6 (17.3)	31.9 (21.0)	15.3 (3.9 – 26.7)	0.010
	37.8	11.9 (13.2)	24.4 (17.4)	12.5 (3.3 – 21.6)	0.009
	38.0	9.4 (10.8)	20.1 (15.3)	10.7 (2.8 – 18.6)	0.009
	38.5	4.8 (5.9)	12.1 (11.0)	7.3 (2.0 – 12.6)	0.008
	39.0	2.0 (2.6)	6.7 (7.2)	4.7 (1.4 – 8.0)	0.006
	39.5	0.5 (0.8)	2.9 (3.9)	2.5 (0.7 – 4.2)	0.006
	40.0	0.03 (0.1)	0.8 (1.6)	0.8 (0.1 – 1.4)	0.026

^1^The mean differences between treatment groups were assessed by student t-test.

^2^P-values of less than 0.05 were considered significant.

^3^FCT (in hour): Fever clearance time (The time from the commencement of ibuprofen or placebo therapy to the time that the temperature fell below and remained less or equal a determined fever threshold until parasite clearance).

SD: standard deviation

^4^FT (in hour): fever time (the time spent at a temperature equal or superior to a determined fever threshold)

^5^AUC_fever _(in °C.hour): Area under the curve with fever (the area covered by a temperature curve at a determined fever threshold).

The AUC_fever _was calculated by use of the trapezoid rule.

Data on all 50 patients were available for the intention-to-treat analysis. There was no significant difference between the two groups in the proportion of patients experiencing any adverse event: seven out of 25 (28%) versus nine out 25 (36%) patients had at least one adverse event in ibuprofen and placebo groups, respectively. One of the adverse events (convulsions 12 h after admission in a child receiving ibuprofen) was classified as a serious adverse event and not due to the study medication. The others were classified as mild or moderate in intensity, unlikely to be due to the study drug. These included vomiting, headache, abdominal pain, fatigue, diarrhea, cough, fever, and conjunctivitis.

## Discussion

This study demonstrated that ibuprofen is effective in reducing the time that a child with malaria is exposed to febrile body temperatures. The effect on fever clearance is less obvious and depends on the definition of the fever threshold. This study failed to show a significant effect of ibuprofen in clearing fever in malaria. However, it does demonstrate that the drug is effective in reducing the time that a child is exposed to febrile body temperatures. This difference is due to the fact that the effect of ibuprofen on body temperature lies in the reduction of high fever peaks. In this context, the term "fever peak" signifies elevated temperatures over a period of time, rather that just a temperature maximum.

This study also demonstrated that the area under the fever curve was significantly smaller for children who took ibuprofen compared to those who took placebo. Ibuprofen can reduce the "burden" of fever during malaria. This may procure some kind of comfort to the child. However, it is recognized that the assessment of the child's comfort may be difficult.

Previous studies from Gabon have shown that neither paracetamol nor metamizol had an effect on fever clearance time compared to mechanical methods [5,6]. Naproxen, which is closely related to ibuprofen, both belonging to the propionic acid group, had a small effect on fever clearance time and could reduce fever peaks [6] as observed in the present study with ibuprofen. Compared to paracetamol, ibuprofen has been shown to be more efficacious for the treatment of fever with infectious diseases in children [7-11]. Studies comparing the two drugs in malaria showed conflicting results: Ibuprofen was shown to be more effective in Thai adults [12], but equally effective in Malawian children [13].

The use of antipyretics in febrile children remains controversial. For the comfort of the child and, most of the time, to satisfy parents' requests, physicians or other health care professionnels are often obliged to prescribe antipyretic treatments to combat fever, although there is no evidence of the therapeutic benefit of this treatment. Moreover, it has been shown that fever may be beneficial for the patient [18]. A number of animal studies have shown various beneficial effects of fever: the survival value of fever in the desert iguana infected with *Aeromonas hydrophila *and the harmful effect of antipyretic treatment in the same animals have been demonstrated. [19,20].

In vitro studies have indicated that a variety of human immunological defence mechanisms function better at febrile temperatures than at normal ones [21]. Interleukin-1 (IL-1) and other endogenous pyrogens have a number of direct effects on the immune response including B-lymphocyte proliferation and antibody production, T-lymphocyte activation, helper T-lymphocyte proliferation, enhanced T-lymphocyte cell killing, and interferon production and function. But, despite the enhanced immune function that occurs at febrile temperatures, studies in humans have not convincingly demonstrated common clinically important risks with the use of antipyretic therapy in common viral and bacterial infections. However, rare serious adverse events occurring after intake of antipyretics remain a concern.

Prevention of "febrile convulsion" is also one of the reasons for a systematic treatment of fever in children. However, there is no evidence to show that antipyretics treatment reduce the risk of febrile convulsion [22-25]. Furthermore, it is likely that not the fever itself, but the systemic pathophysiological changes during infections can induce convulsions [26], fever being only a confounding factor in this relationship. Taken the case of *P. falciparum *malaria as an example, more than half of all convulsions occur at a time point when the children do not have fever (rectal temperature < 38.0°C) [26,27]. Thus, the concept of "febrile convulsions" is questionable. Recent sudies have demonstrated that cellular activation and the consequent release of an inflammatory cytokine cascade may play a role in the pathogenesis of febrile seizures [28,29].

In this study there was no difference in parasite clearance time between the ibuprofen and the placebo group, whereas one might expect a prolonged PCT in the group receiving ibuprofen. This has been shown in previous in vivo study, in which paracetamol prolonged parasite clearance time [5] and in an in-vitro study which showed a strong growth inhibition of *P. falciparum *at 40°C [1].

The present study confirms the results of previous studies in this area, which have shown that antipyretic drugs commonly used (paracetamol, metamizol and naproxen) have no significant effect on fever clearance time. However propionic acid compounds have an effect in reducing fever peaks and reducing the time that a child spends with fever during malaria. This raises questions about the usefulness of antipyretics in children with malaria and other infectious diseases, bearing in mind that all antipyretics even in correct doses can induce serious adverse events, in rare cases even fatal adverse events. Additionally, there is a high risk of over dosing [30]. Moreover the cost for prolonged antipyretic treatment may be substantial for poor families.

Therefore, antipyretics should not be given automatically to all children with fever. Emphasis on the cause rather than the effect of the fever will promote an efficient use of health service and household resources targeted at children who are likely to benefit, and will promote better compliance with essential treatments.

## Authors' contributions

PBM contributed to the study design, data collection, data analysis, interpretation of the results and preparation of the manuscript.

MAM contributed to the study design, data collection, data analysis, interpretation of the results and preparation of the manuscript.

MN contributed to the study design, data collection, and data analysis.

SI contributed to the study design, data collection, data analysis, interpretation of the results and preparation of the manuscript.

BL contributed to the study design, data collection, data analysis, interpretation of the results.

EM contributed to the study design, data analysis and preparation of the manuscript.

PGK contributed to the study design, data collection, interpretation of the results and preparation of the manuscript.

All authors read and approved the final manuscript.

## Conflict of interest

The authors declare that they have no competing interests.
